# Supplementary material for: Knowledge, Attitude, and Practice Regarding Antibiotic Use and Resistance for Upper Respiratory Tract Infections among the Population Attending a Mass Gathering in Central India: A Cross-Sectional Study
Source: Antibiotics (Basel). 2022 Oct 25;11(11):1473. doi: 10.3390/antibiotics11111473 (PMC9686560; doi:10.3390/antibiotics11111473)
Supplement: Supplementary file 1 [file antibiotics-11-01473-s001.zip › antibiotics-1963409-supplementary.pdf]

Supplementary Materials

Knowledge, Attitude, and Practice Regarding Antibiotic Use and Resistance for Upper Respiratory Tract Infections among the Population Attending a Mass Gathering in Central India: A Cross-Sectional Study

Ngoc V. Nguyen [1,2,†], Yogyata Marothi [3,†] and Megha Sharma [1,4,*]

[1] Department of Global Public Health—Health Systems and Policy: Medicines, Karolinska Institutet, 17177 Stockholm, Sweden
[2] Clinical Epidemiology Division, Department of Medicine Solna, Karolinska Institutet, 17177 Stockholm, Sweden
[3] Department of Microbiology, R. D. Gardi Medical College, Ujjain 456006, India
[4] Department of Pharmacology, R. D. Gardi Medical College, Ujjain 456006, India
* Correspondence: E-mail: megha.sharma@ki.se
† These authors contributed equally to this work.

THE QUESTIONNAIRE

Table S1. Socio-demographic information.

No	Question	Response alternatives
1	What is your birth year?	....
2	What is your sex?	1. Male<br>2. Female
3	Where is your place of residence?	1. City<br>2. Village
4	What is the type of your family?<br>*Extended family: a family where grandparents, uncles' family, and cousins live together in one house residing in the same household (a family with three or more generations living together in a household).<br>*Unit family: a family consisting of a married couple with or without their children.	1. Extended family<br>2. Unit family
5	What is your highest-achieved education level?<br>*High school: 10th grade of Indian schooling system	1. Below high school<br>2. High school<br>3. Above High school
6	What is your current occupation?	1. Private job<br>2. Self-employed<br>3. Housewife<br>4. Business<br>5. Student<br>6. Government job<br>7. Others: ....
7	Who is the decision maker of the family?	1. Father<br>2. Mother<br>3. Grandparent

Table S2. Upper respiratory tract infections.

a. History of upper respiratory tract infections.

No	Question	Response alternatives
1	Have you had upper respiratory tract infections before?	1. Yes<br>2. No
2	What do you think, from which source you got the infection?	1. My child<br>2. Another family member<br>3. Working place<br>4. Environment<br>5. Don't know
3	Were you prescribed antibiotic(s) for the recent incident of upper respiratory tract infection?	1. Yes<br>2. No<br>3. Don't know
	Total	3 questions

b. Knowledge of upper respiratory tract infections.

No	Question	Response alternatives
4	What are the most typical symptoms of upper respiratory tract infections?	1. Running nose<br>2. Sore throat<br>3. Cough<br>4. Vomiting<br>5. Ear pain<br>6. Fever<br>7. Anxiety<br>8. Vertigo
5	Is upper respiratory tract infection, a communicable disease?	1. Yes<br>2. No
6	What do you think are the main causes of upper respiratory tract infections?	1. Weather change<br>2. Eating outside<br>3. Eating sour food<br>4. Organisms or germs<br>5. Contact with a sick person<br>6. Allergy<br>7. Pollution<br>8. Don't know
7	Do you know what the causative organism of upper respiratory tract infections is?	1. Bacteria<br>2. Virus<br>3. Fungi<br>4. Parasite<br>5. Don't know
8	Is an antibiotic necessary to treat upper respiratory tract infections?	1. Yes<br>2. No
8.1	If yes, why?	1. Get better faster<br>2. Prevents complications<br>3. Prescription compliance<br>4. Other: ....
8.2	If No, why?	1. Upper respiratory tract infections are self-limiting<br>2. Antibiotics have side effects<br>3. Cost of antibiotics<br>4. Other: ....
	Total	6 questions

Table S3. Knowledge about antibiotic use and resistance.

No	Question	Response alternatives
1	What are antibiotics?	....
2	Do antibiotics have side effects or adverse reactions?	1. Yes<br>2. No<br>3. Don't know about it
3	What can be the consequences of the irrational use of antibiotics?	1. Antibiotic resistance<br>2. More severe disease<br>3. Problems in treating in future<br>4. Don't know
4	Do you think that antibiotics are losing their efficacy?	1. Yes<br>2. No
	Total	4 questions

Table S4. Attitudes towards antibiotic use and resistance.

No	Question	Response alternatives
1	Do you think that antibiotics should be kept as preserved medications for the future?	1. Yes<br>2. No<br>3. Don't know
2	If a doctor does not prescribe antibiotics, will you pressurize him/her to prescribe an antibiotic (s)?	1. Yes<br>2. No<br>3. Sometimes
3	. If a doctor does not prescribe antibiotics, will you go to another doctor who might prescribe an antibiotic (s)?	1. Yes<br>2. No<br>3. Sometimes
4	Who is responsible for the irrational use of antibiotics?	1. Allopathic doctors<br>2. Local non-allopathic practitioners<br>3. Pharmaceutical companies<br>4. Medical shops/Pharmacy patients<br>5. Informal doctors (unlicensed doctors)<br>6. All of the above<br>7. Don't know
	Total	4 questions

Table S5. Attitudes towards antibiotic use and resistance.

No	Question	Response alternatives
1	Have you ever purchased and administered an antibiotic without a doctor's prescription?	1. Yes<br>2. No<br>3. Don't know
1.1	If yes, why did you administer antibiotic(s) without a doctor's prescription and not visit a doctor?	1. Lack of money to pay doctor's fee<br>2. Lack of time to go to a doctor<br>3. Doctors will prescribe the same<br>4. Chemist/Pharmacist knows the treatment<br>5. I know the treatment
2	Have you noticed a red line on some medicine strips?	1. Yes<br>2. Not paid an attention<br>3. Don't know about any such line
3	Would you use any leftover antibiotics or the same prescription to purchase the antibiotics whenever you have similar symptoms with previous infections?	1. Yes<br>2. No
4	Do you purchase the complete antibiotic (s) course written in the prescription by the doctor?	1. Yes<br>2. No, only purchase a few days of the prescribed course. I will purchase more if I will not feel well
5	Do you comply to the entire course of the prescribed antibiotic (compliance to administer prescribed antibiotic (s) for the complete duration)?	1. Yes<br>2. No
5.1	If No, why?	1. Side effects<br>2. Symptomatic relief obtained<br>3. To cut the cost<br>4. Change of doctor<br>5. Other: ....
	Total	6
